# Developmental and quantitative expression profile of the six pollen allergens of mugwort (*Artemisia vulgaris* L.)

**DOI:** 10.1186/s12870-025-06917-9

**Published:** 2025-07-02

**Authors:** Agata Frątczak, Ewa M. Stein, Anna Kasprowicz-Maluśki, Łukasz Grewling

**Affiliations:** 1 Department of Systematic and Environmental Botany, Faculty of Biology, Adam Mickiewicz University, Uniwersytetu Poznańskiego 6, Poznań, 61-614 Poland; 2 Present Address: Department of Molecular and Cellular Biology, Faculty of Biology, Adam Mickiewicz University, Uniwersytetu Poznańskiego 6, Poznań, 61-614 Poland; 3 Laboratory of Aerobiology, Faculty of Biology, Adam Mickiewicz University, Uniwersytetu Poznańskiego 6, Poznań, 61-614 Poland; 4 Present Address: Center for Advanced Technologies, Adam Mickiewicz University, Uniwersytetu Poznańskiego 10, Poznań, 61-614 Poland

**Keywords:** Mugwort, Allergen, Gene expression, Pollen allergy, Anther, Pollen development

## Abstract

**Background:**

*Artemisia vulgaris* L. (Asteraceae family), a wind-pollinated perennial weed, is a significant source of allergenic pollen, responsible for respiratory allergies in late summer. Six allergenic proteins—Art v 1, Art v 2, Art v 3, Art v 4, Art v 5, and Art v 6—have been identified in *A. vulgaris* pollen. However, knowledge regarding significant scientific questions, such as where, when, and in what quantities these proteins are expressed, remains limited.

**Results:**

This study fills these gaps by determining the expression profiles of all six genes encoding allergenic proteins in mugwort pollen. The real-time PCR method was used to analyze the level of allergen expression at three stages of pollen development: microsporocytes before meiosis (stage I), tetrads after meiosis (stage II), and enclosed mature pollen (stage IIIa), as well as isolated mature pollen grains (stage IIIb). The results showed that the expression levels of the most immunogenic allergens, Art v 1 and Art v 3, are extremely high at stage IIIa but very low at stage IIIb, suggesting their production occurs in mature inflorescence tissues. The expression levels of these two major allergens are significantly higher than those of other minor allergens in *Artemisia* pollen. Art v 2 is expressed in both pollen grains and anther tissues, whereas Art v 5 and Art v 6 are transcribed only in mature pollen, with no noticeable expression in earlier stages of pollen development. Art v 4 expression begins at the tetrad stage and reaches its highest levels in mature pollen grains, where it surpasses the expression level of all other allergens.

**Conclusions:**

Our study provides new insights into allergen expression in *A. vulgaris* pollen, highlighting significant quantitative and developmental differences. These findings may help explain why some proteins are more likely to cause pollen allergies than others.

## Background

Pollen hypersensitivity stands as one of the leading causes of IgE-mediated allergies, affecting over 30% of the global population, with this number expected to rise in the forthcoming decades [1–3]. The common mugwort (*Artemisia vulgaris* L.) is the significant source of pollen allergens worldwide. It is a wind-pollinated perennial weed belonging to the Asteraceae family, whose pollination season typically occurs in late summer [4–7]. The prevalence of mugwort allergy in Europe among sensitized patients is estimated to be around 15% on average, reaching a maximum of 45% in Hungary [8, 9]. In China, the positive skin prick responses to *Artemisia* pollen among patients suffering from asthma and/or rhinitis were estimated to be 11.3% [5]. *Artemisia* species also serve as a relevant source of allergens in North America [10]. Additionally, cross-reactions between mugwort and other pollen allergens, e.g. ragweed (*Ambrosia artemisiifolia*), as well as many food allergens such as celery, peach, mustard, or chamomile, are also very common [11–14]. It’s worth adding that *Artemisia* pollen is considered the main vector of airborne endotoxin, which is essential for inducing inflammation of the lung and allergic sensitization [15].

To date, six proteins of *A. vulgaris* pollen have been officially acknowledged as allergens according to the World Health Organization and International Union of Immunological Societies (WHO/IUIS) Allergen Nomenclature Sub-Committee (http://allergen.org/index.php). Art v 1 has been characterized as a major mugwort pollen allergen, with up to 90% of mugwort pollen-allergic patients sensitized to this 28 kDa glycoprotein [16]. The globular amino-terminal domain is cysteine rich and displays a high homology to plant defensin. Notedly, Art v 1 was identified as a defensin-polyproline–linked protein (DPLP). Moreover, this protein, as other plant defensins, is categorized within the pathogenesis-related 12 (PR-12) family [16–19]. Art v 2 has been identified as a glycoprotein and classified into the PR-1 family. It is composed of two identical subunits linked by disulfide bonds with a molecular mass of 19.2 kDa under reduced conditions and 34.2 kDa under non-reduced conditions [20, 21]. Art v 3 is classified as non-specific lipid transfer protein type 1 (nsLTP) and a PR-14 family member. This 12 kDa protein belongs to the widely distributed pan-allergen superfamily of prolamins [22–24]. Art v 4 has been identified as a 14 kDa profilin [25], a cross-reactive pan-allergen which is responsible for multiple food and pollen allergies [26]. It exists as homodimers and tetramers, probably stabilized by sulfhydryl and/or ionic interactions [25]. Art v 5–10 kDa protein belongs to the large family of calcium-binding proteins (CBPs), which are widely distributed pan-allergens. Art v 5 was classified as minor allergen [27]. Art v 6, a 44 kDa member of the pectate lyases family, has been reported to play only a minor role in allergic disease [28].

So far, mugwort pollen allergens have only been partially characterized in terms of their structure (as described above) and localization in pollen grains [29–31]. The biological functions of these proteins remain poorly defined, primarily inferred from their structural properties [32]. Limited information exists regarding the production of allergens in mugwort pollen, including their allergenic activity and IgE binding potencies [33, 34]. A single local quantitative monitoring study has investigated the concentrations of mugwort allergens, specifically Art v 1, in the atmospheric air [35]. Lastly, unlike other pollen allergens such as olive (Ole e 1 and Ole a 3) [36, 37], and birch (Bet v 1) [38], the expression profile of mugwort allergens during pollen development remains unknown. As a result, the existing knowledge about the pollen allergen properties of one of the most potent triggers of inhalant allergy is exceedingly scarce.

Thus, our study addresses this gap by describing the expression profile of all six pollen allergens of *A. vulgaris* during pollen development. To our knowledge, this study represents the first comprehensive analysis of the expression profile of all identified mugwort pollen allergens. We posit that such investigations are particularly important from the perspective of the developmental biology of pollen grain and the functional properties of allergenic proteins. Additionally, understanding the expression patterns of genes encoding specific allergens may shed light on their immunological properties, potentially leading to diagnostic and therapeutic applications.

## Methods

### Sample preparation

**Fig. 1:**
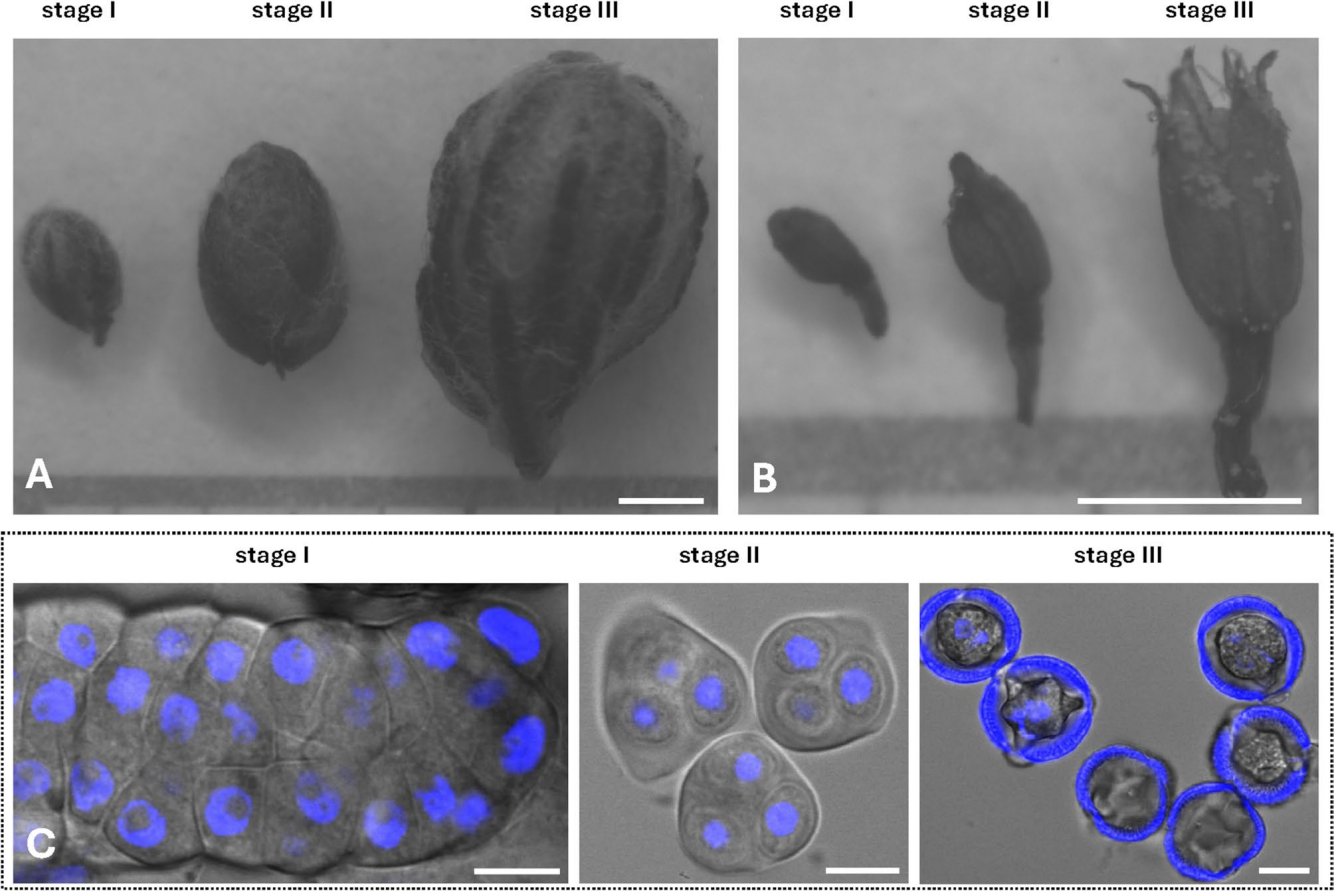
Stages of inflorescence and flower development and corresponding stages of pollen grain development. Stages of inflorescence development (**A**) and corresponding to them stages of flower development (**B**) containing the subsequent stages of pollen grain development. Pictures taken under a binocular. Confocal images of corresponding stages of pollen development (**C**). (I) microsporocytes before meiosis, (II) tetrads after meiosis (III) mature pollen. The scale bar corresponds to 1 mm (**A**, **B**) and 10 μm (**C**). Nuclei stained by DAPI

Inflorescences of *Artemisia vulgaris* L. were collected during the full flowering phase (July–August) from several stands in the northern part of Poznań, Western Poland. The voucher specimens have been preserved in the Natural History Collections in the Herbarium of the Department of Systematic and Environmental Botany (formerly Department of Plant Taxonomy) of Adam Mickiewicz University in Poznań (POZ), under the specimen numbers POZ-V-0169042, POZ-V-0169043, POZ-V-0169044 and POZ-V-0169045, respectively. The botanical specimens were identified by Agata Frątczak. All materials used in this study comply with international and national legal standards. Flowers were examined with Zeiss SteREO Lumar.V12 binocular. Pollen grains were classified into three developmental stages: microsporocytes before meiosis (Stage I), tetrads after meiosis (Stage II), and mature pollen (Stage III) (Fig. 1). Stage III was further divided into two substages: Stage IIIa, containing mature pollen grains within the inflorescences, and Stage IIIb, consisting of pure pollen isolated from flowers (cleaned by double sieving with mesh diameters of 100 μm and 71 μm). The pollen developmental stages were confirmed under Nikon A1 R confocal microscope, with a x40 magnification objective. Nuclei were stained with 6 µg/mL DAPI, fluorescence was excited with 405 nm laser and emission detected with 450/50 nm filter and brightfield images of specimens were captured simultaneously. The collected biological material was immediately frozen in liquid nitrogen and stored at −80 °C.

### Actin as the best reference gene

Special attention was given to the selection of a robust housekeeping gene which is constitutively and equally expressed in all examined stages of pollen grains development. Therefore, based on published sequences from related species *Artemisia annua* and *Artemisia californica*, several candidates were tested: elongation factor 1-alpha (GenBank FJ874734.1), glyceraldehyde-3-phosphate dehydrogenase (GenBank GQ870632.1 and JN411819.1), ubiquitin-conjugating enzyme (GenBank EF549585.1) and actin 1 (GenBank EU531837.1). Initially, using *Artemisia vulgaris* plant material collected by us as a matrix, we amplified the longest possible fragment of a gene and then we tested primer pairs amplifying fragments optimal to real-time PCR (GenBank PQ879984, PQ879983 and PQ879985). We tested several combinations of primers pairs for each gene. For elongation factor 1 we could not amplify any fragment. In the case of glyceraldehyde-3-phosphate dehydrogenase and ubiquitin-conjugating enzyme, their expression levels were not comparable between different stages of pollen grain development. For our experiments, we finally chose actin with primer pairs Fb and Rb (Table 1) generating an amplicon of 209 bp.

### RNA isolation

Total RNA was isolated using modified phenol-chloroform method. Samples (frozen in liquid nitrogen) were pulped in ice-cold mortar. 1.5 mL of Trizol mixture (contained 10 mL Trizol (38% v/v phenol saturated with 0.1 M sodium acetate of pH = 5.0, 0.8 M guanidine thiocyanate, 0.4 M ammonium thiocyanate and 5% v/v glycerol), 100 µL EDTA (Sigma) and 500 µL of 10% N-Lauroylsarcosine (Sigma)) was added. After 5 min RT incubation, 300 µL of chloroform was added. Sample was shaken and left in RT for 3 min. After 10 min centrifugation (4 °C, 12 000 rpm) aqueous phase was collected and 400 µL of phenol-chloroform (1:3) was put in the sample, which had been centrifuged for 3 min. Phenol-chloroform rinse was repeated, the third time only chloroform was added.

After third centrifugation the top, aqueous phase was transferred to new microtube. Five hundred µL of isopropanol and 500 µL of 0.8 M trisodium citrate and 1.2 M NaCl solution were added in order to precipitate RNA. After 5 min RT incubation, sample was centrifuged for 15 min. Supernatant was removed and the remaining pellet was washed with 70% ethanol three times (first time in the volume of 500 µL, than with 150 µL). Each time the sample was centrifuged for 2 min. RNA pellet was dried for 30 min in 37 °C, then it was dissolved in ultrapure water. The concentration was measured with NanoDrop spectrometer. Quality of isolated RNA was tested using Agilent 2100 Bioanalyzer (Agilent Technologies). There was 200 ng of total RNA applied in the sample, tests were performed according to producer’s recommendation using RNA 600 Nano Kit (Agilent Technologies) according to the producer’s protocol. Only RNA whose RIN was not lower than 7 was used for subsequent stages of analysis. The integrity of RNA was also checked in 2% agarose gel electrophoresis.

### RNA preparation

To remove DNA remnants, the total RNA was treated with DNase I (TURBO DNA free Kit, Ambion). Then a reverse transcription reaction was performed with Maxima First Strand cDNA Synthesis Kit (Thermo Scientific), using 500 ng RNA extracted from pure pollen and inflorescences (containing pollen at three development stages), according to protocol and producer’s recommendation attached to the kit.

### Real-time PCR analysis

During real-time PCR experiments, the fluorescent dye SYBR Green, that intercalates with double-stranded DNA, was used for the detection of the threshold cycle (Ct). Hence, real-time PCR reactions were done using DyNAmoColor Flash SYBR Green Master Mix (Thermo Scientific). Primers for all six *Artemisia vulgaris* pollen allergens used in the experiments were designed based on allergens mRNA sequences and the mRNA sequence of the actin gene, which served as a reference gene. All primers sequences used in the experiment are listed in Table 1. Primers were designed keeping all recommendations described for real-time PCR [39]. The primer pairs were located in regions with the greatest possible sequence homology between all isoforms of the tested allergens available in the GenBank as well as those received by us (GenBank PQ793175, PQ793176, PQ879982, PQ879976, PQ879973, PQ879972, PQ879971, PQ793177, PQ810948, PQ879983).

A standard curve was used to determine the measured values for each gene. It was prepared by performing a qPCR reaction on several 10-fold dilutions of PCR product of the tested gene fragment, the concentration of which was known. Ten microliters volume real-time reactions contained 2x DyNAmo Master Mix (Thermo Scientific), 0.5 µM of each primer and 10 ng of cDNA. There were performed 4 biological and 4 technical repeats for each sample and 4 non template controls for each primer pair. Control reactions on materials after application of DNase I were also used to exclude the presence of genomic DNA from the sample.

All qPCR experiments were run on Bio-Rad CFX96 cycler under following thermal conditions: initial denaturation step at 95 °C for 5 min, 40 cycles at 95 °C for 10 s, 60 °C for 30 s. To ensure that a single product was formed, a melting curve analysis was performed. To obtain melting curves, samples were heated from 60 °C up to 95 °C by increasing the temperature for 0,5 °C every 5 s. The specificity of the product was also tested in 2% agarose gel electrophoresis.

### Data processing and statistical analysis

Experimental data was analyzed using Bio-Rad CFX Manager software and Microsoft Office Excel. All allergen genes were normalized against the actin gene which was used as a reference. All obtained results are expressed in gene copies. To investigate the statistical differences between allergen expression level (gene copy number) detected in all selected developmental phases, the analysis of variance (ANOVA) and post-hoc HSD Tukey tests have been applied. All statistical analysis has been performed using R Statistical Software (v4.3.2; R Core Team, 2023). The graphical presentation of the results were performed in R using ggplot library [40].

**Table 1:** Sequence of primers used for real-time PCR

Product size (bp)	Primer name	Sequence (5’−3’)
209	Actin_F	GCAGAGCGGGAAATTGTGAG
	Actin_R	AGCAGCTTCCATTCCGATCA
102	Artv1_F	ATTCGGGTAAGTGCGACAAC
	Artv1_R	AACTTTCTTTGCCGGCTTCT
170	Artv2_F	GTGGCTTGATGAGAGACTCG
	Artv2_R	TACCGGGAGGGTCATAATTG
97	Artv3_F	AACAAGGATCTCAAATCCGA
	Artv3_R	CCTTGTTGCAATCAGTTTCC
116	Artv4_F	GCTGTCATTCGTGGAAAGAA
	Artv4_R	ACAACCATGTTGCATTGACC
103	Artv5_F	TTTGACGAAGCTTGGCTCTG
	Artv5_R	TTCAGCGAACTCATCATAAGAAATG
196	Artv6_F	GAACGTACGCCATTGGTGGT
	Artv6_R	TCGCACCCTGATGCTACAAA

## Results

### *Art v 1* and *Art v 3* are highly expressed in mature inflorescences

**Fig. 2:**
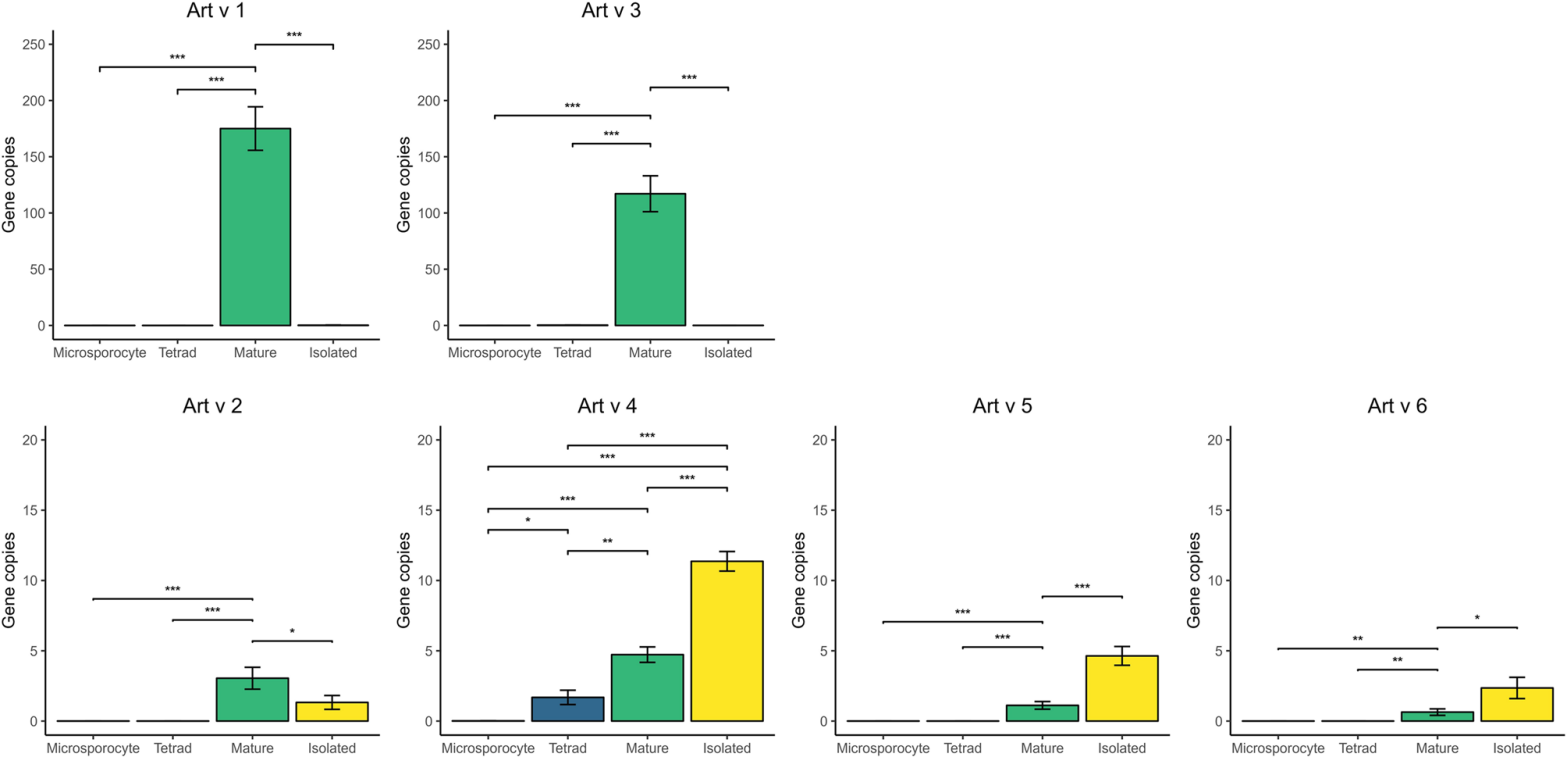
Normalized mRNA expression levels of *Artemisia vulgaris* pollen allergens determined by real-time PCR. Expression levels are normalized against actin. Statistical significance is marked by “*“ (*p* < 0.05), “**” (*p* < 0.01), and “***” (*p* < 0.001). The level of gene expression was analyzed at three developmental stages: microsporocytes before meiosis (Microsporocyte, stage I), tetrads after meiosis (Tetrad, stage II) and mature pollen (stage III). Stage III was analyzed in two variants: IIIa (mature), containing mature pollen grains in inflorescences, and stage IIIb (isolated), consisting of pure pollen isolated from flowers

*Art v 1* and *Art v 3* exhibit very similar expression profiles, with the highest gene copies observed at Stage IIIa. The normalized expression level of *Art v 1* and *Art v 3* reached 175.06 and 117.11 gene copies, respectively (Fig. 2), and was the highest among all investigated stages and allergens. The expression levels of *Art v 1* and *Art v 3* genes in the Stage I and II were negligibly low (between 0.0 and 0.31). Notably, in isolated mature pollen grains, only very low levels of gene copies were recorded (0.23 and 0.08 respectively).

### *Art v 2* is transcribed both in pollen and inflorescence tissues

*Art v 2* was not transcribed during the early stages of pollen development. At the microsporocyte and tetrad stages, gene copy numbers were extremely low (0.001 and 0.004, respectively). Similarly to *Art v 1* and *Art v 3*, the highest expression level of *Art v 2* was observed at Stage IIIa (3.04 gene copies). In pure isolated pollen (Stage IIIb), the *Art v 2* expression level reached 1.32 gene copies, which was markedly higher than that of the two major allergens, *Art v 1* and *Art v 3* (Fig. 2).

### *Art v 4* is gradually expressed during inflorescence development

*Art v 4* shows a distinct expression profile. Similarly to all the investigated allergens, its expression level during the microsporocyte stage was negligible (0.008 gene copies). However, unlike the other allergens, it showed clear expression as early as the tetrad phase (1.68 gene copies). Additionally, at Stage IIIa, the number of gene copies significantly increased (*p* < 0.01), reaching 4.73. Finally, in isolated mature pollen, *Art v 4* reached its highest expression level (11.36 gene copies). None of the other allergens tested showed such high levels at Stage IIIb.

### *Art v 5* and *Art v 6* are late pollen genes exclusively expressed in mature pollen grains

The expression profiles of the *Art v 5* and *Art v 6* proteins were remarkably similar. No mRNA encoding these genes was observed at the microsporocyte and tetrad stages, while the highest gene copy numbers were detected in isolated mature pollen grains (4.64 and 1.42, respectively). In Stage IIIa, expression levels were approximately 3–4 times lower than in Stage IIIb (*p* < 0.05), reaching 1.11 gene copies for *Art v 5* and 0.63 gene copies for *Art v 6*.

## Discussion

In this study, the temporal and quantitative expression of genes encoding all six identified allergenic proteins of *Artemisia* pollen, i.e. Art v 1, Art v 2, Art v 3, Art v 4, Art v 5, and Art v 6, has been determined. To the best of our knowledge, this is the first study to describe the expression profile of all known pollen allergens in a certain plant species.

### *Art v 1* and *Art v 3* are highly expressed in mature inflorescences

Art v 1 and Art v 3 are considered the most allergenic pollen proteins of mugwort [17, 32]. The number of their gene copies in mature inflorescence tissues was extremely high, two orders of magnitude higher than the expression level of any other investigated allergens. For the precision of the results, it should be added that a slight expression of *Art v 3* was also detected in the early stages of pollen development, although it was incomparably lower than in Stage IIIa. Likely, Art v 1 and Art v 3 are secretory proteins, whose expression occurs in the tissues of the anther at the final stage of inflorescence development. Thus, these proteins do not undergo expression in pollen, but are transported to the mature pollen grains from the anther, presumably from its tapetal tissue.

Tapetum, a specialized layer of secretory cells lining the sporogenous tissue of anthers [41], has been already regarded as a source of allergenic proteins for pollen [42, 43]. Tapetum protects pollen grains, provides necessary nutrients, secretes enzymes degrading the tetrad wall, and takes part in the construction of exine, and other specific structures in pollen. During development, pollen grains are immersed in a liquid that conveys nutrients from tapetum. When the pollen is almost ripe, the tapetum disappears, having fulfilled its function [44]. The degradation products of the tapetum transferred to the pollen wall surface form a pollen coat. Numerous nuclear genes show also specific expression in the tapetum [45–47].

**Fig. 3:**
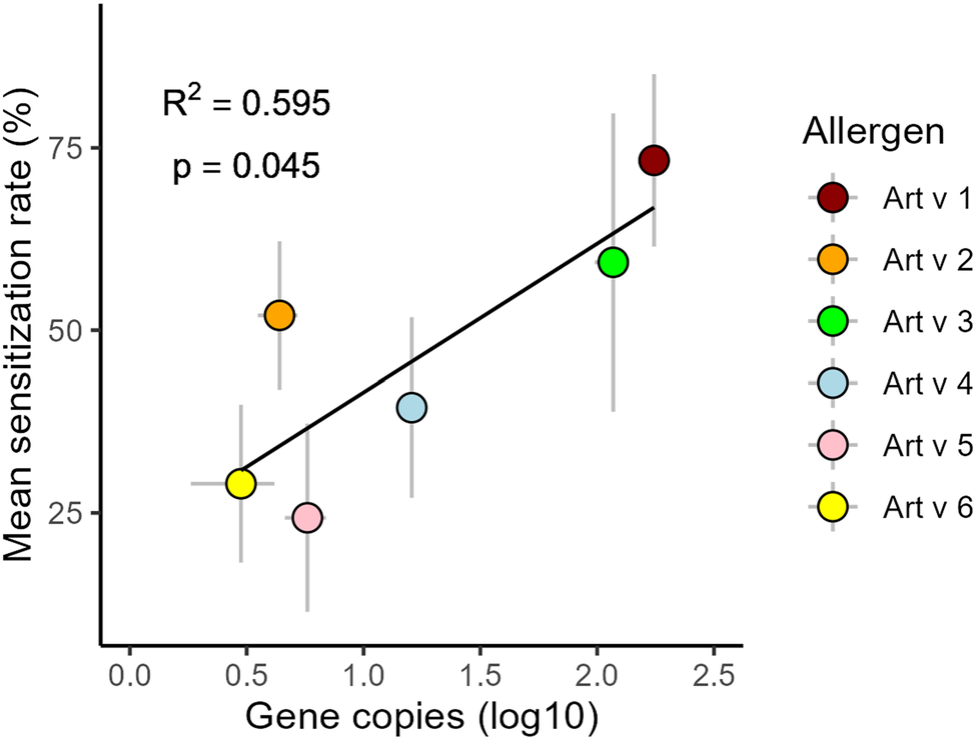
Correlation between mean gene copy number (log10) and mean sensitization rate (%) for six allergens. Note: Sensitization data are based on Table 2, and grey lines indicate standard deviation

The presence of the N-terminal signal sequence in Art v 1 suggests its involvement in the secretory pathway [16], which aligns closely with our findings. Additionally, the tapetal origin of Art v 1 is supported by its predominant localization on the pollen wall surface [29]. In plants, defensin-like pollen proteins, to which Art v 1 belongs, were traditionally believed to be major constituents of the pollen coat [48]. Moreover, it is suggested that these proteins play a significant role in defense against microorganisms [49]. The observation that airborne *Artemisia* pollen is an important vector of bacterial endotoxins [15] may additionally support this assumption. Both extremely high levels of *Art v 1* gene copies detected (in comparison to other investigated allergens) and localization of Art v 1 protein in the pollen coat are undoubtedly significant with respect to allergic sensitization. Art v 1 may rapidly and in high numbers be leached from the pollen wall surface once pollen grains are deposited into the nasal mucosa and then interact with the human respiratory system [29]. This may explain why most *Artemisia* pollen-sensitized patients react to Art v 1 (as well as Art v 3, i.e., another pollen-coat related protein), and not to other examined allergens (Table 2). In this context, the significant correlation (R² = 0.595, *p* = 0.045) between the expression levels of *Artemisia* allergens and the percentage of individuals allergic to specific *Artemisia* allergens is particularly noteworthy (Fig. 3). This observed relationship supports previous findings [50] linking the quantity of produced allergen to its allergenic potential and, consequently, its ability to induce allergic reactions. Although other allergen characteristics, such as localization and solubility [50, 51], should also be considered, the relative amount of allergen in *Artemisia* pollen (expressed here as the level of gene copies) is likely a key factor associated with the sensitization rate to this allergen.

As previously mentioned, Art v 3 has been classified into the family of nonspecific lipid transfer proteins (nsLTPs) [22]. In plants, nsLTPs transport components of the pollen wall from the tapetum to the exine, which is a necessary process for pollen wall development [52]. Our experiment showed that the *Art v 3* gene is transcribed at the highest level in the anther tissues of mature inflorescences (without notable expression in isolated pollen), suggesting its tapetal origin. Thus, the expression profile resembles the pattern described for *Art v 1*. Art v 3 has been predominantly located in the pollen wall, which additionally supports its tapetal origin [29]. It has previously been shown that nsLTPs of other plants, such as *Arabidopsis thaliana*, *Oryza sativa*, and ×*Triticosecale*, also undergo expression in tapetal cells and are then translocated to the pollen cell wall [52–55].

**Table 2:** Sensitization rate (%) to six *Artemisia* pollen allergens. (Note: MAP– mugwort allergic patients, RAP– ragweed allergic patients, NA– data not available)

Allergen	Sensitization rate (%)	Number of patients	Region	Cohort	Test	References
Art v 1	89	9	Austria	MAP	IgE	[56]
	79	100	Germany	MAP	IgE	[57]
	71	17	Italy	MAP	IgE	[28]
	63	27	Poland	MAP	IgE	[13]
	75	24	Spain	MAP	SPT	[17]
	84	50	North Europe	MAP	IgE	[6]
	74	19	South Europe	MAP	IgE	[6]
	46	41	North America	MAP	IgE	[6]
	81	240	China	MAP	IgE	[58]
	59.2	142	China	MAP	IgE	[59]
	73.7	19	NA	MAP	SPT	[21]
	68	34	NA	MAP	IgE	[60]
	73	34	NA	MAP	SPT	[60]
	90	55	NA	MAP	IgE	[16]
Art v 2	45	40	Norway	MAP	IgE	[20]
	42	240	China	MAP	IgE	[58]
	57.9	19	NA	MAP	SPT	[21]
	63.2	19	NA	MAP	IgE	[21]
Art v 3	89	9	Austria	MAP	IgE	[56]
	40	20	Spain	MAP	SPT	[61]
	70.8	24	Spain	MAP	SPT	[17]
	43.7	142	China	MAP	IgE	[59]
	53	240	China	MAP	IgE	[58]
Art v 4	60	9	Austria	MAP	IgE	[56]
	47.5	90	Austria	RAP	IgE	[27]
	34	100	Germany	MAP	IgE	[57]
	41	17	Italy	MAP	IgE	[28]
	20	42	Italy	RAP	IgE	[27]
	29.6	142	China	MAP	IgE	[59]
	47.3	19	NA	MAP	SPT	[21]
	36	100	NA	MAP	IgE	[25]
Art v 5	10	90	Austria	RAP	IgE	[27]
	28	42	Italy	RAP	IgE	[27]
	35	17	Italy	MAP	IgE	[28]
Art v 6	41	17	Italy	MAP	IgE	[28]
	26	NA	NA	NA	NA	[32]
	20	NA	NA	NA	NA	[32]

### *Art v 2* is transcribed both in pollen and inflorescence tissues

In contrast to *Art v 1* and *Art v 3*, obtained results suggest that the expression of *Art v 2* occurs in both mature pollen and anther tissues. The relative expression of *Art v 2* gene copies in mature inflorescences doubled the expression levels in isolated pollen grains. A similar result was obtained for Ole e 1, the major allergen of olive pollen [36]. Two sites of expression of *Art v 2* may explain its localization in different parts of the pollen grain. Art v 2 homologues have been detected in the cytoplasm or in both the pollen wall and cytoplasm, depending on the *Artemisia* species [29]. Based on structural homology Art v 2 was classified to pathogenesis-related PR-1 proteins. In plants, PR-1 proteins are among the most abundantly produced proteins during defense responses constituting 2% of the total leaf proteins in pathogen-infected plants [62]. In our study, the expression level of *Art v 2* in mugwort pollen was however low (3.05 and 1.33 at Stage IIIa and IIIb, respectively), markedly lower than *Art v 1* (app. 175 gene copies), which also belongs to pathogenesis-related protein family (PR-12). The biochemical function of Art v 2 in pollen remains unknown. From clinical point of view, it is suggested that the specific properties of PR-1 proteins, i.e. stability, size, protease resistance, hydrolytic and membrane permeabilizing ability, make them excellent candidates for inducing allergic reactions [63]. The relatively high sensitization rate to Art v 2 among allergic patients, ranging from 42 to 63%, supports this hypothesis [21, 58] (Fig. 3; Table 2).

### *Art v 4* is gradually expressed during inflorescence development

The expression profile of the *Art v 4* gene, which encodes profilin, differs from that of other allergens. Its expression begins as early as the tetrad stage (Stage II) and gradually increases with pollen grain development. In mature pollen grains, the level of its transcripts is nearly seven times higher than at stage II. Additionally, *Art v 4* is the gene with the highest number of transcripts detected in isolated pollen among all examined genes. Art v 4 is a profilin, a protein that binds to actin and plays a role in fundamental cellular processes such as cytoskeleton organization, signal transduction, and pollen tube growth [64]. Thus, it is not surprising that it is already expressed in early stages of pollen development. A similar expression profile to *Art v 4* has also been observed in the pollen-specific profilin of tomato [65]. In other plants, such as maize, lily, and tobacco, profilins were however expressed mainly in mature pollen grains [66–68]. The homologue of Art v 4 in *Platanus acerifolia* (Pla a 2) was located in the pollen cell wall, Golgi bodies, and endoplasmic reticulum, supporting its pollen specificity [69]. It is worth adding that pollen profilins are an important cause of allergy, with prevalence varying between 20 and 30% among people suffering from pollen allergy [27]. In the case of mugwort, this value could be even higher (see Table 2). Finally, due to their high sequence identity and similar folding, profilins show extensive cross-reactivity. As a result, Art v 4-sensitized patients may experience allergy reactions upon contact with different tree and weed pollen grains, as well as various vegetables, fruits, and seeds [11, 64, 70, 71].

### *Art v 5* and *Art v 6* are late pollen genes exclusively expressed in mature pollen grains

Genes *Art v 5* (encoding polcalcin) and *Art v 6* (encoding pectate lyase) exhibited a unique expression profile, supporting their classification as late pollen genes, which are exclusively expressed in mature pollen grains (whose transcripts appear after the first mitotic division of the microspore) [72, 73]. Although their gene copies were detected in both Stage IIIa and Stage IIIb, we suspect that the observed gene expression in Stage IIIa is more likely linked to the ongoing expression in mature pollen (present inside anthers) rather than in the anther tissue itself (e.g. tapetum). This is further emphasized by a markedly lower expression level (approximately 4 times lower) in Stage IIIa compared to Stage IIIb. Polcalcins are proteins that are involved in the control of intracellular calcium levels during pollen germination and in the signaling processes in the pollen tube growth [74]. Thus, their production is justified at the final stage of pollen maturity, as revealed here for Art v 5. Polcalcins are highly cross-reactive allergens detected in various allergenic plant species, such as *Betula pendula* (Bet v 3, Bet v 4), *Phleum pratense* (Phl p 7), *Chenopodium album* (Che a 3), *Olea europea* (Ole e 3, Ole e 8), and *Fraxinus excelsior* (Fra e 3) [74, 75]. They are pollen-specific (not detected in other plant tissues, such as leaves and fruits), located in various parts of pollen grains, e.g. pollen wall, endoplasmic reticulum, mitochondria, and around the nucleus [76, 77]. Similarly as *Art v 5*, the *Ole v 3* transcripts and protein products were only observed in pollen tissue [78]. In contrast, transcripts of Ca^2+−^binding pollen proteins of *Brassica rapa* (Bra r 1 and Bra r 2) have also been detected in earlier stages of pollen development (e.g., the microspore) and in the tapetum [79].

Pectate lyases, which include Art v 6, play a role in tissue remodeling and in pollen tube growth [80, 81]. The expression of pectate lyases has also been observed in mature pollen grains of some other plants, leading to their classification as pollen-specific transcripts [80, 82]. Pectate lyase Cry j 1 from *Cryptomeria japonica* pollen was located in the cell wall, Golgi bodies, and orbicules [83–85]. It is worth mentioning that pectate lyases represent major allergens in almost all pollen grains where they have been identified (Amb a 1, Cup a 1, Jun a 1, Cry 1) [86]. For instance, more than 90% of ragweed-sensitized subjects react to Amb a 1 in skin prick tests and at least 90% of the allergenic activity in ragweed pollen can be attributed to this protein [56]. It was hypothesized, that the incomparably lower sensitization rates to Art v 6 in mugwort-allergic patients (Table 2) might be a consequence of the very low expression levels of Art v 6 in the pollen [86]. Indeed, our results showed that the number of gene copies of *Art v 6* is the lowest among all examined mugwort allergens (Fig. 3).

## Conclusions

The expression profiles of genes coding six *Artemisia vulgaris* pollen allergens have been presented. Temporal (microsporogenesis stages), spatial (pollen vs. tapetum), and quantitative differences in expression levels were observed. Two mugwort allergens, Art v 1 and Art v 3, expressed in the tapetum and later transported to the pollen surface, showed significantly higher expression levels than other allergens. This may explain why these two proteins are considered the primary cause of allergy to *Artemisia* pollen.

It is noteworthy that studies investigating the expression of genes encoding allergenic proteins are relatively underrepresented in the literature. The number of studies in this field remains limited, and several important allergenic taxa have been scarcely explored. This is surprising, as such data can provide important biological insights into the origin and fate of allergens, as well as the functional roles of these proteins. Furthermore, as demonstrated by our results, the quantitative nature of these analyses allows for the assessment of the potential allergenic properties of proteins. Compared to other methods, such as immunoenzymatic assays, gene expression studies offer significant advantages: they are cost-effective, more sensitive, less time-consuming, and do not require specific antibodies, making them broadly applicable. These characteristics render gene expression analysis an ideal tool for large-scale comparative studies of allergens. For example, as Table 2 shows, there is a substantial geographical variation in sensitization rates to specific *Artemisia* allergens. This variation raises the intriguing hypothesis that these differences may stem from variations (both quantitative and qualitative) in allergen profiles among locally dominant *Artemisia* species. Analysis of allergen gene expression represents a promising approach for testing such hypotheses and uncovering the underlying biological mechanisms. Nevertheless, it is important to keep in mind the limitations of gene expression analysis based solely on quantitative mRNA measurements. Real-time PCR reflects transcriptional activity but does not directly quantify protein levels, which is the ultimate factor determining allergenic potential. In this context, complementary methods—such as mass spectrometry-based proteomic analysis—can provide valuable insights. Where financial and technical resources allow, incorporating proteomic approaches into allergen research offers a more comprehensive understanding of allergen composition and enables validation and enrichment of transcriptomic data.

## Data Availability

The datasets used and/or analysed during the current study are available from the corresponding author on reasonable request.
